# The OPVI trial – perioperative hemodynamic optimization using the plethysmographic variability index in orthopedic surgery: study protocol for a multicenter randomized controlled trial

**DOI:** 10.1186/s13063-015-1020-7

**Published:** 2015-11-04

**Authors:** Marc-Olivier Fischer, Georges Daccache, Sandrine Lemoine, Benoît Tavernier, Vincent Compère, Christophe Hulet, Chems Eddine Bouchakour, Christophe Canevet, Jean-Louis Gérard, Lydia Guittet, Emmanuel Lorne, Jean-Luc Hanouz, Jean-Jacques Parienti

**Affiliations:** Pôle Réanimations Anesthésie SAMU/SMUR, CHU de Caen, Avenue de la Côte de Nacre, CS 30001, F-14 000 Caen, France; EA 4650, Université de Caen Basse-Normandie, Esplanade de la Paix, CS 14 032, F-14 000 Caen, France; Service d’Anesthésie Réanimation, CHRU de Lille, Hôpital Roger Salengro, Rue Emile Laine, 59 037, Lille, France; Service d’Anesthésie Réanimation, CHU de Rouen, Hôpital Charles Nicolle, 1 rue de Germont, 76 031, Rouen, France; Department of Orthopedic Surgery, CHU de Caen, Avenue de la Côte de Nacre, CS 30001, F-14 000 Caen, France; Service d’Anesthésie, Hôpital Saint Philibert, 115 rue du Grand But, F-59462 Lomme, France; Department of Public Health, CHU de Caen, Avenue de la Côte de Nacre, CS 30001, F-14 000 Caen, France; INSERM1086, Faculty of Medicine, Caen University Hospital, Avenue de la Côte de Nacre, F-14032 Caen, France; Anesthesiology and Critical Care Department, Amiens University Hospital, Place Victor Pauchet, F-80 054 Amiens, France; INSERM ERI12, Jules Vernes University of Picardy, 12 rue des Louvels, F-80 000 Amiens, France; Department of Biostatistics and Clinical Research, CHU de Caen, Avenue de la Côte de Nacre, CS 30001, F-14 000 Caen, France

**Keywords:** Anesthesiology, Hemodynamic, Orthopedic surgery, Plethysmographic variability index

## Abstract

**Background:**

Hemodynamic optimization during surgery is of major importance to decrease postoperative morbidity and length of hospital stay. However, conventional cardiac output monitoring is rarely used at the bedside. Recently, the plethysmographic variability index (PVI) was described as a simplified alternative, using plug-and-play noninvasive technology, but its clinical utility remains to be established.

**Methods/design:**

The hemodynamic optimization using the PVI (OPVI) trial is a multicenter randomized controlled two-arm trial, randomizing 440 patients at intermediate risk of postoperative complications after orthopedic surgery. Hemodynamic optimization was conducted using either the PVI (PVI group) or conventional mean arterial pressure (control group). The anesthesiologist performed the randomization the day before surgery using an interactive web response system, available 24 hours a day, 7 days a week. The randomization sequence was generated using permutated blocks and stratified by center and type of surgery (knee or hip arthoplasty). Patients and surgeons, but not anesthesiology staff, were blinded to the allocation group. The primary outcome measure is the length of hospital stay following surgery. The attending surgeon, who was blinded to group assessment, determined hospital discharge. Secondary outcome measures are theoretical length of hospital stay, determined using a dedicated discharge-from-hospital checklist, postoperative arterial lactate level in the recovery room, postoperative troponin level, presence of serious postoperative cardiac complications, and postoperative acute kidney insufficiency.

**Discussion:**

The OPVI trial is the first multicenter randomized controlled study to investigate whether perioperative hemodynamic optimization using PVI during orthopedic surgery could decrease the length of hospital stay and postoperative morbidity.

**Trial registration:**

ClinicalTrials.gov NCT02207296.

**Electronic supplementary material:**

The online version of this article (doi:10.1186/s13063-015-1020-7) contains supplementary material, which is available to authorized users.

## Background

While more than 200 million surgeries are performed worldwide each year [1], recent data showed that perioperative morbidity and mortality remain significant [2]. Perioperative hemodynamic complications frequently occur after noncardiac surgery and increase the mortality risk [3].

Perioperative hemodynamic optimization is of major importance to decrease myocardial injury after noncardiac surgery [4], and is recommended in Britain and France [5, 6]. Initially described using complex methods, such as oxygen delivery [7], the concept was simplified to a maximization of stroke volume using titrated fluid loading with cardiac output monitoring [5, 6]. However, although such a strategy is beneficial for patients, it is rarely used at the bedside [8]. The invasive cardiac output monitoring method, a lack of knowledge, and time constraints could partially explain this disappointing result [8]. The prediction of fluid loading using cardiopulmonary interaction could be another method of fluid optimization. Initially described using respiratory arterial pulse pressure variations under mechanically ventilation [9], a noninvasive alternative has recently been described using respiratory variations of the plethysmographic wave form [10].

The plethysmographic variability index (PVI) is determined using an automated, plug-and-play, totally noninvasive device (Masimo Corporation, Irvine, CA, USA) that consists of a simple oximetry sensor connected to a monitor (Radical 7, Masimo Corporation, Irvine, CA), which responds to the simplified criteria needed at the bedside. Previous studies reported that PVI could accurately predict fluid responsiveness [11, 12], and that a forehead sensor could be better than a digital sensor [13]. However, only a few Phase III studies with heavy limitations were conducted, to assess the clinical utility of PVI in decreasing perioperative morbidity [14–16], and the benefit remains to be established.

The OPVI study aims to compare the effects of PVI using perioperative hemodynamic algorithm optimization and a conventional hemodynamic algorithm using mean arterial pressure in patients with intermediate risk of postoperative complications after orthopedic surgery.

## Methods/design

### Ethics and study design

**Fig. 1:**
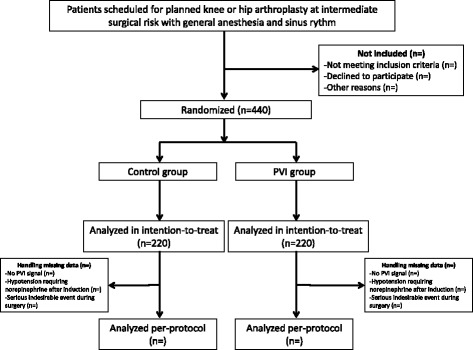
CONSORT diagram for the OPVI trial. PVI, plethysmographic variability index

The hemodynamic optimization using the PVI (OPVI) study is a multicenter randomized controlled two-armtrial. The institutional review board of the University Hospital of Caen approved the study for all co-investigator centers (Registration number ID RDB: 2014-A00330-47, 23 May 2014). The OPVI study is conducted in accordance with the Declaration of Helsinki, and was registered on 31 July 2014 on the ClinicalTrials.gov website with trial identification number NCT02207296. The OPVI trial follows the CONSORT statement [17]; the CONSORT diagram is shown in Fig. 1.

### Study population

Local investigators screen consecutive patients scheduled for planned hip or knee arthroplasty in participating centers. Patients receiving general anesthesia are eligible for the study.

Patients fulfilling one or more of the following criteria will not be included: lack of informed consent prior to randomization, cardiac arrhythmia, sepsis, use of another form of hemodynamic monitoring as cardiac output monitoring, chronic kidney disease with dialysis, black skin (owing to limitations in plethysmography technology), pregnant, younger than 18 years or under judicial protection. This is to provide good technical conditions for the PVI and a relatively homogeneous study population for interpretation of the results.

Included patients were at moderate surgical risk, considering the surgical procedure or the medical history for each patient. Patients at high surgical risk, according to medical history, for whom a cardiac output monitor could be used [6], will be not included in the study. All patients are asked for written informed consent, as required by the institutional review board, in accordance with the Declaration of Helsinki.

### Randomization

Randomization is performed by the anesthesiologist the day before surgery using an interactive web response system with Clinsight® software (Ennov, Paris, France), which is available 24 hours a day, 7 days a week. The randomization sequence is generated using permutated blocks and stratified by center and type of surgery (knee or hip arthoplasty).

### Interventions

After hemodynamic stabilization following general anesthesia induction and orotracheal intubation, included patients are assigned to either the control group or the PVI group, according to the randomization.

**Fig. 2:**
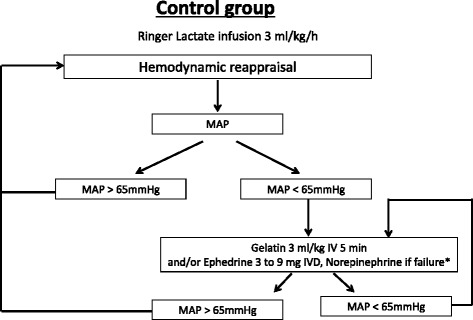
Hemodynamic algorithm for the control group. *Norepinephrine after failure of the use of ephedrine defined by the use of 30 mg of ephedrine without desired hemodynamic response. Norepinephrine: dosage begins at 0.05 μg/(kg min), and is then adjusted in steps of 0.05 μg/(kg min). IV, intravenous; IVD, intravenous drip; MAP, mean arterial pressure

In the control group (Fig. 2), the hemodynamic goal is a mean arterial pressure > 65 mmHg; the clinician could prescribe intravenous fluid challenge using 3 ml/kg of gelatin for 5 minutes or a vasopressor (ephedrine until 30 mg, and norepinephrine after), or both.

**Fig. 3:**
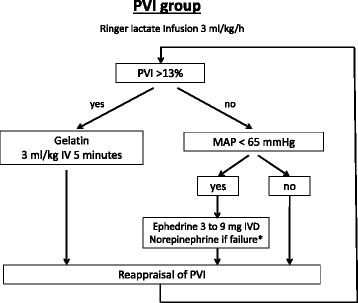
Hemodynamic algorithm for the PVI group. *Norepinephrine after failure of the use of ephedrine defined by the use of 30 mg of ephedrine without desired hemodynamic response. Norepinephrine: dosage begins at 0.05 μg/(kg min), and is then adjusted in steps of 0.05 μg/(kg min). IV, intravenous; IVD, intravenous drip; MAP, mean arterial pressure; PVI, plethysmographic variability index

In the PVI group (Fig. 3), the hemodynamic targets are both the PVI and the mean arterial pressure: the fluid challenge prescription depends on a PVI value > 13 %, according to a previous study [13], while vasopressor use depends on a mean arterial pressure < 65 mmHg.

Both the control group and the PVI group will undergo PVI monitoring, which is continuously recorded from induction of general anesthesia to the end of surgery, but the monitor is blinded in the control group. All PVI data will be recovered by an independent investigator not involved in the patient anesthesia, after surgery. The allocated therapy is delivered until the patient is discharged from the operating room.

### Standard procedures

At arrival in the operating room, each patient undergoes the usual monitoring, including scope, noninvasive blood pressure, and pulse oximetry, until discharge from the recovery room.

The concomitant use of regional anesthesia, choice of anesthetic, prophylactic antibiotics, and postoperative pain management are left to the discretion of the attending anesthesiologist.

The ventilator patterns indicated by the investigators are a tidal volume using 8 ml/kg of ideal body weight, a respiratory frequency and a fraction of inspired oxygen (FiO_2_) according to a range of end tide carbon dioxide (etCO_2_) between 35 and 45 mmHg, and a peripheral capillary oxygen saturation (SpO_2_) > 96 %, respectively.

### Study endpoint measures

The study endpoint measures are listed in Table 1.

**Table 1:** Study endpoint measures

	Endpoint measure	Judgment criteria
Primary endpoint measure	Postoperative length of hospital stay	Real postoperative length of hospital stay (days)
Secondary endpoint measures	Theoretical postoperative length of hospital stay	Theoretical postoperative length of hospital stay using a checklist (days)
	Postoperative arterial lactate level in recovery room	Proportion of patients with lactate levels > 2 mmol/l
	Postoperative troponin level at days 1 and 3	Proportion of patients with troponin Ic > 0.06 ng/ml
	Serious postoperative cardiac complications	Proportion of patients with at least one of: cardiac arrest, arrhythmia, and heart failure requiring treatment
	Postoperative kidney insufficiency	Proportion of patients with an increase of at least 30 % in creatinine level compared with preoperative value

The primary outcome measure is the real length of hospital stay (in days) following planned hip or knee arthroplasty under general anesthesia.

The secondary outcome measures are theoretical length of hospital stay using a dedicated discharge-from-hospital checklist (see Additional file 1) [18], the postoperative arterial lactate level in the recovery room, the troponin level on postoperative days 1, and 3, whether there are any serious postoperative cardiac complications (at least one of the following criteria: cardiac arrest, arrhythmia, or heart failure requiring a treatment), and the postoperative acute kidney insufficiency (defined as an increase of at least 30 % of creatinine compared with the preoperative level [19]). Abnormal values of lactate and troponin levels are beyond 2 mmol/l and 0.06 ng/ml, respectively.

### Blinding

A coding list will be generated using the interactive web response system and each patient from the specific trial site will be allocated a coding number. During surgery and postoperative care, both surgeons and patients are blinded to the allocated group. The material used in the PVI group and in the control group is similar, in keeping with the blinded design. Therefore, patients in each group remain indistinguishable. Only the anesthesiology staff and the research staff can view the monitor (which is blinded in the control group) and know the group allocation. The surgeons are the postoperative care providers, and will decide the length of hospital stay, while remaining totally blinded to the group allocation. The research staff will use the objective checklist for hospital discharge twice a day, at 8 a.m. and 4 p.m. for each included patient.

### Intention-to-treat analysis

Patients with severe hypotension following induction of general anesthesia and requiring norepinephrine, or who experience serious undesirable events during the surgery, justifying invasive hemodynamic monitoring (arterial catheter, cardiac output monitoring), will not be treated using the study group algorithm allocation. They will undergo different monitoring or treatment at the discretion of the attending anesthesiologist, but they will be analyzed according to their initial assigned group on an intention-to-treat analysis.

### Sample size estimation

Using the French national database, two groups of 193 patients are required to detect a difference of 1 day in the primary outcome measure between groups, using a two-sided *α*-risk at 0.05 and a *β*-risk at 0.20, assuming a standard deviation of ±3.5 days for the primary outcome difference. An interim analysis is planned after enrollment of the first 200 patients, to check the length of hospital stay after surgery in each group and perform a futility assessment with conditional power. We anticipate no missing data for the primary outcome measure (length of hospital stay following surgery). However, we plan to conduct multiple imputation in the case of missing data. For per-protocol analysis (sensitivity analysis), the need to handle missing data (failure of PVI monitoring; hypotension following induction of general anesthesia leading to a requirement for norepinephrine; or serious undesirable events during the surgery) will be anticipated by including 27 additional patients for each group. Nevertheless, all randomized patients will be analyzed in the allocated group for the main, intention-to-treat, analysis. In addition, to comply with the intention-to-treat analysis, missing data for the primary outcome will handled by multiple imputation (PROC MI in SAS) and analyzed in sensitivity analyses using PROC MIANALYSE in SAS version 9.4 (SAS institute, Cary, NC, USA).

### Statistical plan

Categorical variables will be described as percentages; continuous variables will be described as mean (with standard deviation) or median (interquartile range), as appropriate. The analysis for the primary outcome will follow the intention-to-treat principle, in which all the randomized patients will be analyzed in the assigned group. The principal comparison will be performed by a multivariate linear regression of the LOS, including the group and stratification factors (center and type of surgery) as independent variables. The normal distribution of the primary outcome will be tested by the Kolmogorov–Smirnov test. Logarithmic transformation will be conducted before analysis in the case of significant departure from the normal distribution. Categorical variables will be compared between groups using the Fisher exact test or the Pearson chi-square test for heterogeneity. All statistical analysis will be conducted using SAS version 9.4 (SAS institute, Cary, NC, USA). Statistical significance will be assumed for *P* < 0.05.

### Registration

Data will be collected and registered using electronic case report forms in each center by a dedicated local technical research team. A research coordinator will centralize data from all sites.

### Data collected and registered

Baseline characteristics and prerandomization data will be recorded: sex, age, height, weight, ideal body weight, Lee score [20], smoking status, history and type of diabetes mellitus, dyslipidemia, history of cardiovascular disease (systemic hypertension, ischemic heart disease, valvular heart disease, peripheral vascular disease, cardiac medications), history of respiratory disease (asthma, chronic obstructive pulmonary disease) using the PROVILHO study criteria [21], history of hepatic disease (Child Pugh classification of cirrhosis [22]), renal insufficiency (classified according to the glomerular filtration rate [23]), history of neoplasia (active or remission), and blood sample (creatinine, bilirubin, albumin, rate of prothrombin).

During the anesthesia and surgical procedures, the following will be recorded: type of procedure (first or reoperation), surgical site (hip or knee arthroplasty), duration of anesthesia and surgery, blood loss and transfusion requirements, all drugs used during anesthesia (anesthetics, opiates, neuromuscular blocking agent), all administered fluids (number of titrated fluid loading and total fluid loading), and all vasoactive drugs.

After the procedure and before the extubation, the blood lactate level will be recorded. Data from the Radical-7 monitor will be extracted (mean PVI, percentage of time that PVI < 13 %, mean heart rate). The mean arterial pressure, the ratio of mean arterial pressure to heart rate, and the percentage of time that the mean arterial pressure is less than 65 mmHg and less than 55 mmHg, will be extracted from the conventional monitor.

During postoperative days 1, 3, and 5, blood samples will be taken, to assess creatinine, troponin, and hemoglobin levels.

From postoperative day 0 until discharge from hospital, any postoperative complications will be recorded (Table 1).

The real and theoretical (using the checklist) hospital length of stay and the survival status at day 30 following inclusion will be recorded.

### Record keeping

Consent forms and electronic case report forms will be stored for 15 years in each center, in accordance with French law.

### Study organization

The study promotion is performed by the University Hospital of Caen, France. There is neither industrial financial support nor industrial involvement in the study protocol.

### Duration and timeline

Patients from five French university hospitals (Caen, Amiens, Lille, Rouen) will be included during a two-year inclusion period, beginning in February 2015.

The protocol, approval from the ethical committee, financial support, electronic case report forms, and interactive web response system were developed in 2014. Inclusion of patients is planned for 2015 and 2016. The database will be closed in 2017, after which data analysis, manuscript writing, and submission for publication will follow.

## Discussion

Perioperative hemodynamic complications frequently occur after noncardiac surgery and increase the risk of mortality [3]. Although perioperative hemodynamic optimization is of major importance to decrease myocardial injury [4], it has rarely been applied [8]. The OPVI trial is the first randomized controlled multicenter study powered to investigate the PVI as a noninvasive hemodynamic tool in patients scheduled for orthopedic surgery.

The primary endpoint measure of the trial is the real length of hospital stay after planned hip or knee arthroplasty. This endpoint measure depends on the perioperative morbidity, and will reveal the clinical impact of a hemodynamic algorithm using PVI. The definition of the main outcome measure is challenging [24], but it seems more appropriate and relevant in evaluating the clinical utility of PVI at the bedside. Moreover, the sample size could be calculated using a minimal hospital length of stay, in practice equal to 3 days, rather than 0 days, which could introduce statistical bias. For example, the sample size calculation for a difference of 1 day between the control group (5 days) and the PVI group (4 days) could correspond to a decrease in hospital length of stay equal to 20 %, if calculated from the first day, or of 50 %, if calculated from the third day, which corresponds to the minimal length of stay observed in practice. However, the standard deviations are larger in this simulation and could decrease the interest of this calculation. As a secondary objective, we evaluate the theoretical hospital length of stay using an objective evaluation with a specified checklist used by dedicated research staff (see Additional file 1) twice a day, at 8 a.m. and 4 p.m. for each included patient. The other secondary endpoint measures determine: (1) oxygen debt, using the blood lactate level in the recovery room, which represents an immediate postoperative prognostic value [25]; and (2) postoperative troponin level [3], cardiac complications [26], or kidney insufficiency [27], which have both short- and long-term prognostic value.

Patients requiring high-risk surgery were not selected for inclusion in study population, because PVI hemodynamic monitoring is considered for intermediate surgical risk [6]. This population of patients is the more frequently observed in practice [2]. Inclusion criteria were large for the study population, reinforcing the external validity of the study.

Some comments could be addressed concerning the limitations of the study. First, the study population was restricted to patients requiring orthopedic surgery with intermediate surgical risk. Further studies could be developed with other types of surgery that have intermediate surgical risk. Second, the medico-economic of PVI use is not evaluated in the present study, but an ancillary study is proposed, to follow it.

In conclusion, the OPVI trial is a multicenter controlled randomized trial, powered to test the hypothesis that perioperative hemodynamic optimization using the PVI algorithm could decrease the length of hospital stay after orthopedic surgery in patients with intermediate surgical risk. The OPVI trial also evaluates the impact of the PVI algorithm on postoperative cardiac complications (at least one of the following criteria: cardiac arrest, arrhythmia, and heart failure requiring treatment), postoperative troponin level, incidence of postoperative kidney insufficiency, and postoperative blood lactate level.

## Trial status

The trial is ongoing and is actively enrolling patients.

## Additional file

Additional file 1:
**Discharge-from-hospital checklist.** All items must be checked ‘yes’ for the patient to be considered for discharge at the time of checklist completion (8 a.m. or 4 p.m.); this time serves to quantify the theoretical length of hospital stay. (DOCX 67 kb)

## Abbreviations

OPVI: optimization using the plethysmographic variability index
PVI: plethysmographic variability index
