# Supplementary material for: The OPVI trial – perioperative hemodynamic optimization using the plethysmographic variability index in orthopedic surgery: study protocol for a multicenter randomized controlled trial
Source: Trials. 2015 Nov 4;16:503. doi: 10.1186/s13063-015-1020-7 (PMC4634899; doi:10.1186/s13063-015-1020-7)
Supplement: Additional file 1: — Discharge-from-hospital checklist. All items must be checked ‘yes’ for the patient to be considered for discharge at the time of checklist completion (8 a.m. or 4 p.m.); this time serves to quantify the theoretical length of hospital stay. (DOCX 67 kb) [file 13063_2015_1020_MOESM1_ESM.docx]

**Additional file 1 Discharge from hospital checklist**

|  | YES | NO |
| --- | --- | --- |
| Conscious and adapted patient: can give the current date and address |  |  |
| Hemodynamic stability: blood pressure and heart rate did not differ +/- 20% of preoperative values |  |  |
| Afebrile |  |  |
| Has no difficulty breathing and has a SpO2 > 92% ambient |  |  |
| Has no nausea or vomiting for 24 hours |  |  |
| Normally feeds |  |  |
| Natural urinary and gases |  |  |
| Can walk alone with canes or walkers |  |  |
| Last hemoglobin value > transfusion threshold* |  |  |
| Pain controlled (AVS < 3/10) with oral analgesics |  |  |
| Scar clean and not disunited |  |  |

All items must be checked « yes » for the patient to be considered out of service during time completion (8AM or 4PM) then serving to quantify the theorical length of hospital stay.

*transfusion threshold [18]:

10 g.dL-1: patients with cardiopulmonary disease showing signs of intolerance.

8 g.dL-1 patients to be active and limited in their activities, as well as those with a history of cardiovascular or older than 65 years.

6 g.dL-1: transfusion usually indicated unless there is a causal treatment (pernicious anemia, iron deficiency anemia, anemia of chronic renal failure) or if the tolerance is acceptable.
